# Compensatory Social Networking Site Use, Family Support, and Depression Among College Freshman: Three-Wave Panel Study

**DOI:** 10.2196/18458

**Published:** 2020-09-02

**Authors:** Mingjie Zhou, Fugui Li, Yanhong Wang, Shuang Chen, Kexin Wang

**Affiliations:** 1 CAS Key Laboratory of Mental Health Institute of Psychology Chinese Academy of Sciences Beijing China; 2 Department of Psychology University of Chinese Academy of Sciences Beijing China; 3 Mental Health Counseling Center Yang-En University Quanzhou China; 4 School of Media University of Chinese Academy of Social Sciences Beijing China; 5 College of Media and International Culture Zhejiang University Hangzhou China

**Keywords:** freshmen, introversion, compensatory use of SNS, depression, family support, social media

## Abstract

**Background:**

Freshmen were found to use social networking sites (SNS) as a useful medium to effectively adjust to college life, which hints at a tendency to resort to SNS for social compensation. However, the compensatory use of SNS is usually problematic.

**Objective:**

This study explores why a subgroup of freshmen developed depressive symptoms while socially adjusting to college by investigating the antecedent role of introversion, the explanatory role of compensatory use of SNS, and the protective role of perceived family support. The study is among the first to point out the relevance of the compensatory use of SNS in explaining the indirect association between introversion and depression with a longitudinal design.

**Methods:**

A 3-wave panel sample of freshmen (N=1137) is used to examine the moderated mediation model.

**Results:**

We found that introversion at Wave 1 positively predicted compensatory use of SNS at Wave 2 and subsequently increased depression at Wave 3 (unstandardized B=0.07, SE 0.02, *P*<.001, 95% CI 0.04-0.10; unstandardized B=0.09, SE 0.01, *P*<.001, 95% CI 0.06-0.12). The moderated mediation model further examined the buffering role of perceived family support within the link between introversion and compensatory SNS use (index=0.0031, SE 0.0015, 95% CI 0.0003-0.0062). Unexpectedly, we found that family support in Wave 1 decreased compensatory SNS use for less introverted freshmen in Wave 2 and further decreased depression in Wave 3.

**Conclusions:**

Unexpectedly, our findings uncover an enhancing effect, rather than a buffering effect, of family support by embedding its effect within the relationship between introversion and compensatory SNS use. Appreciating the differences in the casual pathways for freshmen with different levels of introversion clarifies how SNS affect young adults' lives.

## Introduction

### Freshmen Use of Social Networking Sites and Depression

Matriculating at a university, a process that marks the transition into emerging adulthood, can be a challenging test for many freshmen to adjust to [1]. Among various psychological symptoms that may appear during maladjustment, depression is given particular notice by researchers. For instance, a recent study discovered that 25.5% of freshmen reported depressive symptoms, with total scores higher than 16 on the Center for Epidemiologic Studies Depression (CES-D) scale; moreover, nearly 6% reported suicidal ideation [2]. In college students, depression is associated with suicidal behavior [3], increased risk of substance use [4], as well as reduced retention [5]. Thus, it is of critical importance to identify risk factors and to unravel influential mechanisms in the adjustment phase.

The use of social networking sites (SNS) is identified as a new potential risk factor for depression, especially among adolescents and emerging adults [6]. Despite the fact that SNS provide a new channel for young people to maintain old connections and build new ones, some studies indicate that the problematic use of SNS may harm adjustment [7]. Compensatory internet use, one of many different kinds of problematic internet uses, is closely related to stressful experiences during the first months of college life. Notably, compensatory internet use is related to compensatory use, which was proposed based on the social compensation hypothesis [8], indicating that people who experience difficulties in offline sociality would end up benefiting from using online interaction platforms (eg, chat rooms). Compensatory internet use, focusing more on a motivational perspective, refers to using a broad range of internet applications as a coping strategy for a negative life situation [9].

More specifically, compensatory internet use is conceptualized by Kardefelt-Winther [9] as a fascination with going online to escape real-life problems or attenuate dysphoric feelings; this coping strategy may cause maladaptive outcomes. For instance, Wang and colleagues [10] found that college students under high stress resort to compensatory use. In addition, Elhai et al [11] indicate that compensatory smartphone use, as one specific type of compensatory internet use, co-occurred with various constructs of depression. Moreover, compensatory internet use can be driven by different life difficulties [9]. Among these difficulties, social problems were found to be predominant in compensatory internet use, leading to the creation of the concept of compensatory SNS use (eg, the use of Facebook) [12]. Essentially, compensatory SNS use specifies using SNS as a means of compensation for personal social inadequacy and thus can be seen as a certain type of compensatory internet use. Following this reasoning, this study sought to investigate the association between freshmen's compensatory SNS use and depression during the stressful transition to university life.

However, it is important to note that the tendency to resort to SNS for social compensation varies among individuals. In general, individuals with low levels of social competence (eg, high in introversion) are more likely to use SNS in a compensatory way. Moreover, various studies have found that the structure of social support is pertinent to the coping strategy [13,14]. Family, along with friends and significant others, is an invaluable social support [15]. According to the social support buffering hypothesis, perceived support has been found to have a buffering effect for negative coping in adverse life situations [16,17]. As such, social support may interact with social traits to impact coping strategy and, in turn, influence mental health. Specifically, we investigated the joint effect of introversion and perceived family support on compensatory SNS use and subsequent depression at a later time among freshmen.

Specifically, the study sought to (1) examine the mediating role of compensatory use of SNS within the association between introversion and depression among freshmen at the very beginning of their adjustment to university life, and (2) test whether family support would protect introverts from resorting to compensatory use and subsequently decrease the risk of developing depression. Notably, the study extends prior research in 3 ways: First, by including media-use risk factors for freshmen, we hope to increase existing knowledge about how freshmen cope with the transition to college. Second, by identifying potential vulnerable groups and protective factors, we present a new, highly targeted intervention for freshmen adjustment. Third, we are the first to use 3-wave data to test our hypotheses on a moderated mediation model for introversion, compensatory SNS use, and depression; this approach responds to a scholarly call for longitudinal research on the antecedents for and outcomes of SNS use.

### Introversion and Freshmen's Depressive Symptoms

Scholars believe that core personality is a major concomitant of depression. Introversion (ie, low extroversion), among the Big Five constructs, is especially promising in its association with the phenomenology and the outcome of depression [18]. Various studies have found a positive relationship between introversion and depression. The positive association between introversion and depression might be explained by Eysenck's theory. According to this theory, introverts are more susceptible to punishment [19] or frustrative nonreward [20], which may increase the risk of experiencing a negative mood. In line with this theory, Larsen and Ketelaar [21] found that extroverts reacted more to a positive mood than a negative mood; introverts, however, reacted more to a negative mood than a positive mood. According to Jung's theory [22], introverts are oriented toward internal thoughts and, thus, tend to be ruminative, unsociable, and reserved toward others; this may also explain why more introverted people tend to be more depressed. Empirical evidence from cross-sectional studies supports this positive association. For instance, Saklofske et al [23] found that introversion was positively linked with depression. However, one study using a nationally representative sample found that introversion was related with, but not a significant predictor of, depression [24].

Notably, compared to what was found in cross-sectional studies, the association between introversion and depression in longitudinal studies was weaker. Shull [17] reports a longitudinal association between introversion and depressive symptoms among first-year college students; however, according to a meta-analysis study, the association between introversion and depression across studies mostly remains observed but seems markedly attenuated when controlling for baseline depression levels.

Taken together, since freshmen's negative moods (ie, a combination of anxiety, tension, depression, anger, confusion, fatigue, and a lack of vigor) increase significantly over time [25], it is likely riskier for more introverted freshmen to develop a depressive mood. Empirical studies found that freshmen's social self-efficacy and self-disclosure were protective predictors of depression [26]; rumination, on the other hand, was one reason why more introverted freshmen became more depressed than extroverts during the transitional phase into university [27]. Therefore, based on Eysenck's theory, Jung's theory, and the longitudinal nature of our study, we assumed that introversion would positively—but weakly—predict depressive freshman mood over time (hypothesis 1).

### Compensatory SNS Use as a Mediator

Compensatory SNS use, or the tendency to “go online to escape real-life issues” [9], has been identified as a risk factor for young people's well-being. For instance, Weidman et al [28] suggest that using the internet as an alternative to face-to-face communication reduces well-being. In addition, a few studies indicate a positive association between SNS addiction and depression, which shares an interrelated psychological process with compensatory SNS use [12] and depression [29]. This negative impact of compensatory SNS use on young people's mental health can be explained by Compensatory Internet Use Theory [9], which argues that “the locus of the problem is a reaction by the individual to his negative life situation, facilitated by an internet application.” For instance, when young people encounter situations where social stimulation is lacking, they prefer to turn to an internet application (ie, to SNS) that makes socializing accessible instead of making an effort to socialize with the people around them. This escapist coping strategy may have a short-term positive effect in that it can help users get their desired reward of sociability. However, it leads to detrimental effects in the long run: Users may come to rely solely on the internet for socializing [12].

Compensatory SNS use may be particularly salient among freshmen during their college transition. On the one hand, freshmen are unique in that they experience a drastic disruption in their social networks as their families and old friends are, to some extent, out of reach when they attend college. At the same time, they are new to college and have not made significant social ties. On the other hand, the popularity of SNS and smartphones makes it unprecedentedly effortless for freshmen to compensate for sociality online (eg, by turning to an old friend or making new friends online) in contrast to socializing with a potential friend offline. As a result, it is of great importance for scholars to look into this social adjustment phase by focusing on freshmen's compensatory SNS use. However, freshmen vary in their ability to negotiate college life; previous studies have identified several fundamental traits that may contribute to the success or failure of negotiating college life [25].

Because of the centrality of introversion in determining responses to mood, frustration, and social issues, it is conceivable that freshmen with different levels of introversion might react differently to compensatory use and subsequently show diverse levels of vulnerability to depression. In line with this reasoning, Peter et al [30] argue that introverts, who may have difficulty building friendships in person, are more prone to use online contacts as a substitute for an offline social network. Several studies have examined this proposed association between introversion and compensatory use of the internet. For example, Zywiza and Danowski [31] show that a subset of introverted Facebook users strived to make themselves look more popular through online activities. In addition, as suggested above, Goby [32] found that, compared to extroverts, introverts are more likely to use the internet to expand their social network. Therefore, based on social compensation theory and empirical findings, we assumed that compensatory SNS use would mediate the association between introversion and depression (hypothesis 2).

### Family Support as a Buffer

Social support was found to moderate the relationship between adverse situations and well-being. More precisely, it can act as a buffer to alleviate the negative influence of stress on depression [33]. This buffer depends on both the size and the structure of the social network [13]. Zimet and colleagues [15] conceptualized the structure of a social network by considering the sources of support, namely, family, friends, and significant others, as distinct subgroups. How and when students receive support from these groups, therefore, becomes important in preventing depression. In addition, Kenny [34] found that the stability and value of family ties positively affect freshmen's social well-being. As noted above, freshmen face the challenge of “leaving home and separating from families and friends” [35]. Moreover, although separation does not necessarily mean being cut-off, low family support was found to be responsible for depression among college students; in particular, it notably interacted with their experiences of stress [36].

Buffering hypothesis indicates that social support can protect one from psychological suffering, but the effects are relatively unimportant for those with low levels of stress [37]. For social adjustment, more introverted freshmen experience higher interpersonal stress [38] and inadequacy and thus might be more prone to use SNS for compensation than less introverted freshmen. Based on the buffering hypothesis, more introverted freshmen tend to benefit from family support than less introverted freshmen, as buffering effects are more effective for those with higher stress. In line with this reasoning, Anschuetz [39] found that social support, including family support, has a buffering effect for highly introverted freshmen to improve their social adjustment.

Specifically, social support can help reduce maladaptive coping for vulnerable people [40]. When stressed people perceive adequate instrumental or emotional support, they are less likely to cope with stress adversely. For instance, a recent study reported that social support significantly buffers the association between stress- and coping- motivated alcohol use among college students [41]. As reasoned previously, compensatory internet use generally, and compensatory SNS use specifically, is not a very healthy coping strategy [16], and introversion is positively related to potentially maladaptive compensatory use. Given that social support can reduce such maladaptive coping, we expected family support to have a moderating effect on the relationship between introversion and compensatory SNS by weakening this link. In other words, more introverted freshmen will be less likely to use SNS for a compensatory motive when they have more family support than when they have less family support.

This moderating effect can be explained by the bidirectional theory, which defines coping as constantly changing cognitive and behavioral efforts to balance internal demands and external support [42]. According to this theory, when an individual's current demands exceed their appraised resources, they will cope in negative ways [43]. Compensatory SNS use is mostly conceptualized as a negative coping strategy [9]. As freshmen negotiating college social life, they would also grapple with both external and internal demands, especially for those high in introversion because they may experience a lower level of social competence than those low in introversion. In this sense, family support is an external resource, which may alleviate an introvert's negative coping strategy by reducing compensatory SNS use. Based on such theories and empirical studies, we theorized that family support would moderate the relationship between introversion and compensatory SNS use and subsequently influence depression; specifically, the relationship between introversion and depression will be weaker when family support is high (hypothesis 3).

**Figure 1:**
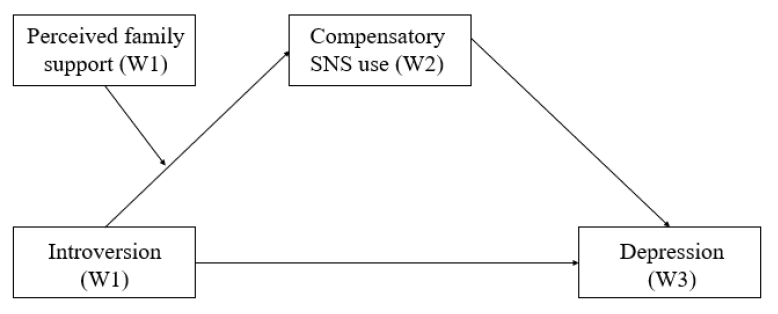
Proposed model of the relationship between introversion (W1) and Depression (W3), with compensatory use of SNSs (W2) as a mediator and family support (W1) as a moderator on this mediating mechanism.

This study explores why a subgroup of freshmen developed depressive symptoms while socially adjusting to college by investigating the antecedent role of introversion, the explanatory role of compensatory use of SNS, and the protective role of perceived family support. Figure 1 depicts our proposed model.

## Methods

### Sample and Procedures

We conducted a 3-wave panel study with an interval of 1 month between each wave, which immediately began after freshmen had registered at a large university (the country has been deleted for peer review) in September 2017. Previous studies of freshmen adjustment have focused on the first 3 months because maladaptive behavior would already be revealed by then [44], and also because the adjustment during this phase can predict further performance.

Trained researchers distributed questionnaires during school hours. Participants were informed that the survey was confidential and that their responses would only be used for research purposes. The researchers obtained informed consent from each of the participants and approval from the host university.

In the first wave, out of 1428 freshmen, 1350 (94.54%) completed the questionnaire. In the second wave, of the 1350 freshmen who had responded in the first wave, 1270 (94.07%) freshmen completed the questionnaire again. In the third wave, of the 1270 freshmen who had participated in the first two waves, 1137 (89.53%) responded to the questionnaire. The final sample consisted of 1137 freshmen, 62.27% (708/1137) of which were women and 37.73% (429/1137) of which were men. The mean age was 18.76 years (SD 0.86). We used a multivariate analysis of variance (MANOVA) to test the potential attribution effects. Using Pillai trace, a MANOVA with all relevant Wave 1 variables showed no significant differences between those who participated at Wave 1 (N=1350) and those who also participated at Wave 3 [N=1137; V=0.01, F(1104)=1.55, *P*=.12, hp^2^ = 0.01].

### Measures

#### Control Variables

Participants reported their age (in years) and gender (1=*male*, 2=*female*). Family income was measured by asking participants to rate their family income level in comparison to the majority of families with whom they were acquainted, using the following scale: *extremely lower than others* (=1), *a lot lower than others* (=2), *a little bit lower than others* (=3), *average* (=4), *a little bit higher than others* (=5), *a lot higher than others* (=6), and *extremely higher than others* (=7). In addition, participants indicated whether they were an only child (*yes=*1, *no=2*) and whether their family lived in the same city as their university (*yes*=1, *no*=2) in the first wave. We also controlled for baseline depression as a covariate in the moderated mediation model.

#### Introversion (Wave 1)

We used the subscale of extroversion from the 10-item Big Five Inventory (BFI-10) [45] to construct a measure of introversion. On a 7-point scale *(disagree strongly*=1 to *agree strongly*=7), participants rated the extent to which they agreed with the following descriptions: “I see myself as someone outgoing and sociable” and “I see myself as someone reserved.” An average score was calculated after recoding the reversed item. Higher scores represented introversion*.* The internal consistency was 0.75.

#### Compensatory SNS Use (Wave 2)

We extracted the subscale of compensatory use of Facebook from the Psycho-Social Aspect of Facebook Use (PSAFU) scale [12]. By replacing “Facebook” with “SNS,” we revised the scale to be more general. The resulting 8-item subscale includes items such as “I have more fun socializing on SNS than in real life” and “I find it easier to communicate with people on SNS than in face-to-face, real settings.” Participants ranked their agreement with these items from *strongly disagree* (=1) to *strongly agree* (=5). The mean value of these responses was calculated as a new variable. The internal consistency of the scale was 0.80.

#### Perceived Family Support (Wave 1)

The subscale of the multidimensional scale of perceived social support (MSPSS) developed by Zimet et al [15] was used to measure support from family. Participants rated their agreement with 4 items (eg, “I get emotional help and support from my family” and “my family really tries to help me”) from *strongly disagree* (=1) to *strongly agree* (=5). An average score was created to represent perceived family support. The internal consistency of the scale was 0.86.

#### Depression (Wave 1 and Wave 3)

The patient health questionnaire (PHQ-9) [46]) was used to assess the severity of depression. Participants rated the frequency of described symptoms (eg, “Feeling that you are a failure or have let yourself or your family down” and “having trouble falling or staying asleep, or sleeping too much”) from *not at all* (=0) to *nearly every day* (=3). A mean score was calculated as an indicator of depression. The internal consistency of the scale was 0.81 in Wave 1 and 0.84 in Wave 3.

### Analytical Strategy

We used the PROCESS macro (model 7) for SPSS (version 21.0, IBM Corp) with bootstrapping (95% CI, 1000 samples) to analyze the data [47]. The model included the following control variables from Wave 1: gender, age, siblings, relative family income, family location, and baseline depression. Table 1 presents the mean, standard deviation, and zero-order correlations among variables.

**Table 1:** Descriptive statistics (N=1137).

Variables^a^	Mean	SD	1	2	3	4	5	6	7	8	9
1.Gender (W1^b^)	—^c^	—	1								
2. Only child (W1^b^)	—	—	.23^d^	1							
3. Family location (W1^b^ )	—	—	-.03	.01	1						
4. Relative family income (W1^b^ )	3.57	0.80	.03	-.07^e^	-.05	1					
5. Age (W1^b^ )	18.76	0.86	-.03	.13^c^	-.02	-.09^c^	1				
6. Depression (W1^b^)	0.62	0.39	.07^d^	.06^d^	-.03	-.08^c^	.02	1			
7. Introversion (W1^b^ )	3.59	1.37	.03	.05	-.07^d^	-.05	-.04	.23^c^	1		
8. Perceived family support (W1^b^)	5.29	1.20	.02	-.06^d^	.01	.12^c^	.03	-.28^c^	-.14^c^	1	
9. Compensatory SNS^f^ use (W2^g^)	2.61	0.73	-.17^c^	-.08^c^	-.02	-.02	-.04	.21^c^	.16^c^	-.17^c^	1
10. Depression (W3^h^)	0.68	0.39	.02	.05	.01	-.05	-.04	.51^c^	.11^c^	-.25^c^	.24^c^

^a^ Numbered row headings correspond with numbered column headings.

^b^ W1: Wave 1.

^c^—: Not applicable.

^d^*P*<.01.

^e^*P*<.05.

^f^SNS: social networking sites.

^g^W2: Wave 2.

^h^W3: Wave 3.

## Results

### Introversion and Depression (Hypothesis 1)

Hypothesis 1 predicted a positive association between introversion (Wave 1) and depression (Wave 3) across time. In contrast with the prediction of hypothesis 1, the results showed that introversion in the first wave was not significantly associated with freshmen's depression in the third wave. Thus, hypothesis 1 was not supported.

### Mediating Role of Compensatory SNS Use (Hypothesis 2)

Hypothesis 2 predicted that compensatory SNS use (Wave 2) would mediate the association between introversion (Wave 1) and depression (Wave 3). More specifically, hypothesis 2 predicted that introverted freshmen would resort to compensatory SNS use more frequently and thus have a higher risk of developing depression compared with extroverted freshmen who may not as frequently resort to compensatory use. In line with hypothesis 2, the results revealed that the association between introversion in the first wave and depression in the third wave was mediated by compensatory SNS use in the second wave. This indicated that compensatory SNS use in the first months explained why freshmen with different baseline levels of introversion gradually developed different levels of depression in the following 2 months. Therefore, hypothesis 2 was supported.

### Perceived Family Support as a Moderator in the Mediating Model (Hypothesis 3)

Hypothesis 3 further posited that perceived family support (Wave 1) would moderate the association between introversion (Wave 1) and compensatory SNS use (Wave 2). More specifically, hypothesis 3 aimed to test whether family support weakens the positive link between introversion and compensatory SNS use by decreasing more introverted freshmen's SNS use.

**Figure 2:**
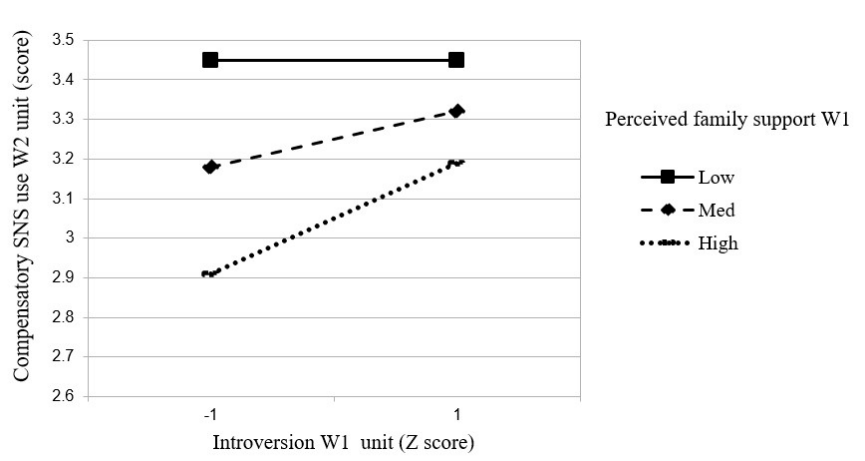
The moderating effect of perceived family support (W1) in the relationship between introversion (W1) and compensatory use of social networking sites (SNS; W2).

Roughly in line with hypothesis 3, the moderated mediation test revealed that perceived family support (Wave 1) was a significant moderator of the relationship between introversion (Wave 1) and compensatory SNS use (Wave 2) (index=0.003, SE 0.001, 95% CI 0.0003-0.0062). To interpret the interaction effect, 3 simple slope tests were conducted. When family support was low (ie, 1 SD below the mean), the influence of introversion on compensatory SNS use was not significant (unstandardized *B*=0.003, SE 0.002, , *P*=.18, 95% CI 0.001-0.007); When the family support was medium, the influence of introversion on compensatory SNS use was significantly positive (*B*=0.007, SE 0. 002, *P*<.001, 95% CI 0.004-0.010). When the family support was high (ie, 1 SD above the mean), the influence of introversion on compensatory SNS use was also significantly positive (*B* =0.010, SE 0.002, *P*<.001, 95% CI 0.006-0.014). Thus, family support can alleviate the impact of introversion on compensatory SNS use only when family support is at medium and higher levels. However, in contrast to the prediction of hypothesis 3, the beneficiaries of family support are less introverted freshmen rather than more introverted freshmen. As depicted in Figure 2, the protective effect of family support on the association between compensatory SNS use (Wave 1) and depression (Wave 3) was stronger for less introverted freshman than for those who were more introverted. As such, hypothesis 3 was not supported.

**Figure 3:**
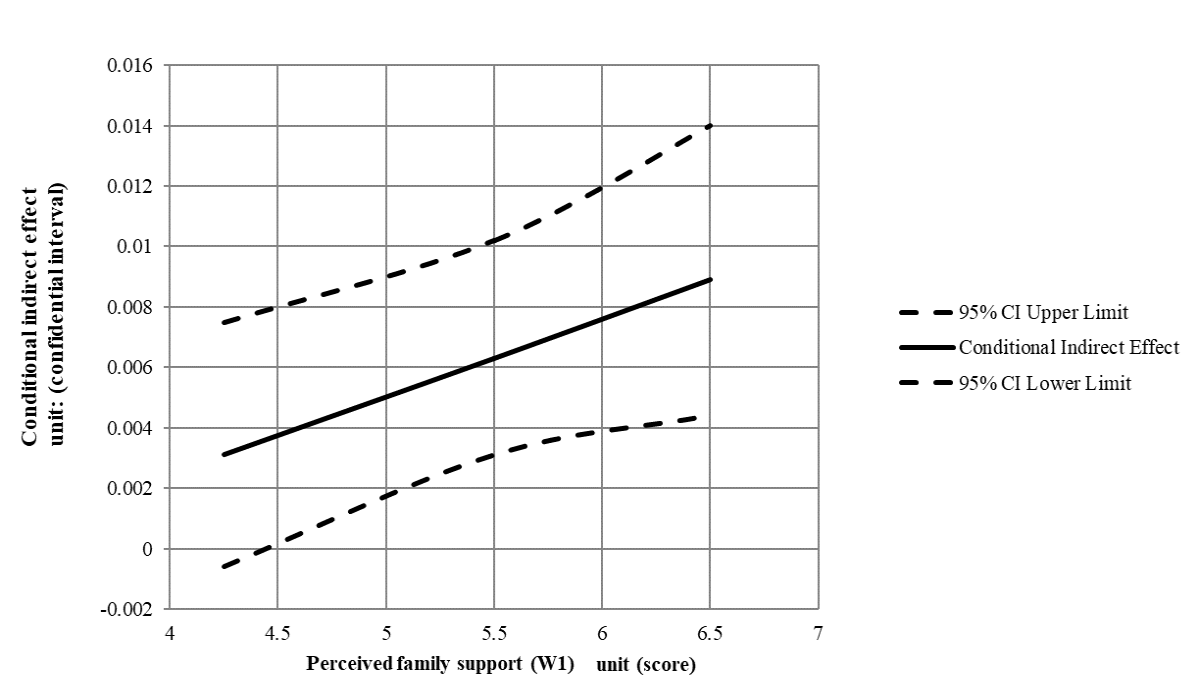
The conditional indirect effect of introversion (W1) on depression (W3) at values of the perceived family support (W1) through compensatory use of social networking sites (SNS; W2).

Table 2, Table 3, and Figure 3 show the results of the moderated mediation analysis. We also conducted the regression analysis with baseline depression as the only control variable. Hypothesis 1 was also not supported (B=-0.01, SE .01, *P*=.25, 95% CI -0.02-0.01), suggesting that introversion at Wave 1 was not a significant predictor of depression at Wave 3. Hypothesis 2 was supported (B=0.09, SE 0.01, *P*<.001, 95% CI 0.06-0.11), suggesting compensatory SNS use at Wave 2 mediated the relationship between introversion at Wave 1 and depression at Wave 3. Moreover, the moderated mediation analysis was also supported (index=0.003, SE 0.001, 95% CI 0.0004-0.0063), indicating that perceived family support at Wave 1 moderated the relationship between introversion at Wave 1 and compensatory SNS use at Wave 2. However, in contrast to hypothesis 3, the results revealed that as perceived family support at Wave 1 increased, a decrease in introversion in Wave 1 was related to a decrease in depression in Wave 3. These focal results were inconsistent with the results reported in Table 2.

**Table 2:** Results of moderated mediation analysis (N=1137).

Predictors	Compensatory SNS^a^ use (W2^b^)				Depression (W3^c^)			
	*B* ^d^	SE	LLCI^e^	ULCI^f^	*B* ^d^	SE	LLCI^e^	ULCI^f^
Constant	3.25^g^	0.49	2.28	4.22	0.50	0.24	0.03	0.98
Gender (W1^h^)	-0.27^g^	0.04	-0.36	-0.18	0.01	0.02	-0.03	0.05
Age (W1^h^)	-0.02	0.02	-0.07	0.02	-0.02	0.01	-0.04	0.00
Only child (W1^h^)	-0.10^j^	0.05	-0.19	-0.01	0.03	0.02	-0.01	0.07
Relative income (W1^h^)	0.01	0.03	-0.04	0.06	0.01	0.01	-0.01	0.05
Family location (W1^h^)	-0.01	0.03	-0.07	0.05	0.02	0.01	-0.01	0.05
Depression (W1^h^)	0.34^g^	0.06	0.23	0.45	0.48^g^	0.03	0.43	0.54
Introversion (W1^h^)	0.07^g^	0.02	0.04	0.10	-0.01	0.01	-0.02	0.01
Perceived family support (W1^h^)	-0.20^g^	0.06	-0.32	-0.09	N/A^i^	N/A	N/A	N/A
Introversion (W1^h^) * Family support (W1^h^)	0.03^j^	0.01	0.01	0.06	N/A	N/A	N/A	N/A
Compensatory SNS use (W2^b^)	N/A	N/A	N/A	N/A	0.09^e^	0.01	0.06	0.12
*R* ^2^	0.11				0.28			
∆*R*^2^	0.11^g^				0.17^g^			

^a^SNS: social networking sites.

^b^W2: Wave 2.

^c^W3: Wave 3.

^d^*B*: unstandardisd coefficient.

^e^LLCI: lower level confidential interval.

^f^ULCI: upper level confidential interval.

^g^*P*<.001.

^h^W1: Wave 1.

^i^N/A: not applicable.

^j^*P*<.05.

**Table 3:** Conditional indirect effects of introversion (Wave 1) on depression (Wave 3) at the value of perceived family support (Wave 1) through compensatory social networking sites use (Wave 2).

Family support	Effect	Boot SE	Boot LLCI^a^	Boot ULCI^b^
-1 SD	0.0031	0.0021	-0.0008	0.0077
Mean	0.0063	0.0018	0.0030	0.0103
+1 SD	0.0089	0.0025	0.0044	0.0139

^a^LLCI: lower level confidential interval.

^b^ULCI: upper level confidential interval.

## Discussion

### Principal Findings

Previous studies have documented that freshmen vary in their capacity to negotiate college social life [48] and that depression rises significantly during this adjustment phase [49]. This longitudinal study sought to increase our knowledge of the association between introversion and depression by investigating the explanatory role of compensatory SNS use and the protective role of perceived family support during the initial phase of transition. We found that introversion at Wave 1 positively predicted compensatory use of SNS at Wave 2 and subsequently increased depression at Wave 3. The moderated mediation model further examined the buffering role of perceived family support within the link between introversion and compensatory SNS use. Unexpectedly, we found that family support in Wave 1 decreased compensatory SNS use for less introverted freshmen in Wave 2 and further decreased depression in Wave 3. This finding indicated that family support served as an enhancing role for freshmen with low introversion rather than a buffering role for freshmen with high introversion.

### Hypothesized Model

In contrast to the prediction of hypothesis 1, we found that introversion was not significantly associated with depression across time. This finding differs from previous research that found support for a positive association between introversion and depression among freshmen [27]. However, our finding is in line with a recent study that also found no significant association between introversion and depression among freshmen in nursing [50]. In addition, another study based on a national representative sample also found that introversion was related to, but not a significant predictor of, depression. More specifically, “introversion may be better viewed as reflective of shared variance with depression” [24]. Furthermore, Cheng and Furnham [51] argued that although introversion was often a direct predictor of happiness, it was never a direct predictor of depression. Together with the finding in hypothesis 2, we provide more evidence on the indirect association between introversion and depression.

In line with hypothesis 2, we found that compensatory SNS use was a significant mediator within the association between introversion and depression, suggesting that compensatory use can explain how freshmen with different levels of introversion developed different levels of depression in their first 3 months of university (after controlling the baseline depression). The positive association between introversion and compensatory SNS use can be explained by the social compensation hypothesis, which posits that individuals with difficulty managing social life are more likely to use online interaction as a substitute [52]. The positive association between compensatory SNS use and depression can be explained by research on escapism. Escapism is a coping strategy that can aggravate current and future depression because it exacerbates people's vulnerability to stressful events by making them feel increasingly helpless, inadequate, and nervous [53]. It is possible that freshmen who use SNS to compensate for socializing become more depressed because they feel increasingly inadequate in sociality. Taken together, the confirmed mediation pathway provides a possible causal mechanism for understanding, from a media-use perspective, how a subset of freshmen become more depressed.

Partially in line with hypothesis 3, we found that perceived family support (Wave 1) was indeed a moderator within the association between introversion (Wave 1) and compensatory SNS use (Wave 2), indicating that family support interacted with introversion to affect freshmen's compensatory SNS use. However, this moderating effect contradicted our hypothesis for a buffering effect. As shown in Figure 2, under medium and high family support conditions, compensatory SNS use increased with introversion but did not exceed the low family support condition. However, when family support is relatively adequate, people low in introversion reported less compensatory SNS use. As people low in introversion are more capable of coping with stressful life events and thus have better mental health, we would argue that family support serves as an enhancing role for people who are low in introversion.

The moderated mediation effect may be explained by differences in orientation preferences between more introverted people and less introverted people. Less introverted people are interpersonally oriented while more introverted people have intrapersonal orientations; this suggests that when external family support is available, people differing in introversion vary in gaining the support. Specifically, less introverted people are more likely to benefit from social support. For instance, Kushwaha [54] found that extroverts seek significantly more guidance and support from others when coping with illness. In addition, Zell et al [55] suggested that extroverts have higher levels of intimacy and trust in their offline social ties than introverts, which may translate into greater benefits and more effective support when needed. Accordingly, when family support is relatively abundant, less introverted freshmen are less likely to perceive themselves as socially inadequate and are thus less likely to use SNS as compensation than more introverted freshmen. However, when external family support is low, freshmen show no difference in using SNS for social compensation, as it seems available and effortless. This enhancing effect was also found in resilience. Although resilience has been frequently theorized as a buffer for more vulnerable groups when facing adverse events [56], one study found that resilience further facilitates well-being among less vulnerable people [57]. Future study may be needed to investigate the boundary condition and the mechanism for both buffering and enhancing effects.

### Implications

This study makes 3 key contributions that may guide future research. First, the results detected compensatory SNS use as a risk factor for depression among freshmen during their adjustment to college—this conflicts with the findings of a previous study that theorizes compensatory use as only the consequence, not the cause, of other psychological problems [9]. Therefore, the results of this study suggest a need for further investigation into the negative effect of compensatory SNS use in other stressful life events among other subgroups. More specifically, it may be highly relevant to uncover why and how certain types of SNS use meant to make people feel better end up being harmful. Second, the results supported the hypothesized mediating role of the compensatory use of SNS within the association between introversion and depression, which clarifies how core personality (introversion) predicts well-being (depression). Previous studies posit that extroversion is a direct predictor of happiness but not depression [51]. Our findings may thus increase understandings of the mechanism by which introversion may indirectly predict depression. Third, our findings reveal that family support has an enhancing effect for freshmen with low introversion to avoid using SNS out of a compensatory motive in the first 3 months of adjustment; this indicates the need for further investigation into how family support functions for vulnerable youth using negative coping strategies, especially during key developmental phases.

However, some questions still remain unanswered. First, if family support plays a role in preventing compensatory SNS use and thus decreases depression over time, it may be highly relevant to investigate whether other sources of social supports (eg, friendship) also reduce maladaptive SNS use and, in turn, decrease depression. In addition, it remains unclear why family support did not prevent depression in more introverted freshman. It could be that such freshman resorted to compensatory use for self-affirmation or recognition from sources other than social supports; that is, social contact itself may not have satisfied this group's inner need for validation. We hope this study can spark future researchers to take up these questions.

Notably, in practical terms, the study revealed one possible mechanism through which freshmen become more depressed at the very beginning of their enrollment: More introverted freshmen are likely to resort to compensatory SNS use, which increases depression over time. When family support is relatively sufficient, freshmen low in introversion reported less compensatory SNS use and lower levels of depression than those high in introversion. This may help university administrators and families gain insight into prediction and targeted intervention for vulnerable freshmen.

### Limitations

When interpreting our findings, 2 limitations have to be considered. First, the measurement of compensatory SNS use adopted in this study does not differentiate between various kinds of SNS. However, the type of SNS may influence the psychological outcomes of compensatory use. For instance, WeChat, the most prevalent SNS in China, is a relatively closed platform on which freshmen have more acquaintances or even intimate contacts than in other platforms; in contrast, QQ, which is especially popular among young adults, involves a larger percentage of strangers and superficial contacts. Consequently, seeking social compensation through WeChat may not aggravate depressive symptoms as much as seeking compensation through QQ. Further research should consider the differences between these platforms, perhaps by applying a more nuanced measurement of compensatory use.

Second, although our findings provide insight into the potential casual mechanism of how freshmen become more depressed by investigating the explanatory value of compensatory SNS use, the study remained focused on media and sociality. Other moderators (eg, perceived friend supports) and mediators (eg, self-reaffirmation) should be examined in future studies to provide a more comprehensive picture of how freshmen adjust to academic achievement and other aspects of college [58].

### Conclusion

In today's highly mediatized environment, young adults have access to an ever-accumulating set of SNS. Socially vulnerable freshmen may use online contacts as a substitute for face-to-face interactions, especially when their transition to college disrupts their social networks. The current study is among the first to point out the relevance of the compensatory use of SNS in explaining the indirect association between introversion and depression with a longitudinal design. Our findings uncover an enhancing effect of family support by embedding its effect within the relationship between introversion and compensatory SNS use. Together, appreciating the differences in the casual pathways for freshmen with different levels of introversion clarifies how SNS affect young adults' lives.

## Abbreviations

MANOVA: multivariate analysis of variance
SNS: social networking sites
